# Use of intrauterine dextrose as an alternative to systemic antibiotics for treatment of clinical metritis in dairy cattle: a microbiome perspective

**DOI:** 10.3389/fvets.2024.1478288

**Published:** 2024-12-16

**Authors:** Jennine Lection, Emily Van Syoc, Asha Miles, Julia Hamilton, Marcela Martinez, Santiago Bas, Justin Silverman, Adrian Barragan, Erika Ganda

**Affiliations:** ^1^Intergraduate Degree Program in Integrative and Biomedical Physiology, Huck Institutes of the Life Sciences, The Pennsylvania State University, University Park, PA, United States; ^2^Department of Animal Science, College of Agricultural Sciences, The Pennsylvania State University, University Park, PA, United States; ^3^One Health Microbiome Center, The Pennsylvania State University, University Park, PA, United States; ^4^Department of Biology, Eberly College of Science, The Pennsylvania State University, University Park, PA, United States; ^5^Animal Genomics and Improvement Laboratory, Agricultural Research Service, United States Department of Agriculture, Beltsville, MD, United States; ^6^Department of Veterinary and Biomedical Sciences, College of Agricultural Sciences, The Pennsylvania State University, University Park, PA, United States; ^7^Phytobiotics Futterzusatzstoffe GmbH Bvd Villa Maria Córdoba Argentina, Villa Maria, Argentina; ^8^College of Information Sciences and Technology, The Pennsylvania State University, University Park, PA, United States; ^9^Department of Medicine, The Pennsylvania State University, Hershey, PA, United States; ^10^Institute for Computational and Data Science, The Pennsylvania State University, University Park, PA, United States

**Keywords:** antibiotic alternative, clinical metritis, dairy cattle, intrauterine dextrose, microbiome

## Abstract

**Introduction:**

Clinical metritis (CM) has significant costs to dairy producers. Current treatment strategy involves systemic antibiotics; however, there is increasing concern about judicious antibiotic use. The study objective was to evaluate the effects of a non-antibiotic treatment vs. systemic antibiotic therapy on the vaginal discharge microbiome of dairy cows diagnosed with CM at 7 ± 3 DIM (days in milk). We hypothesize that both treatment methods will have a similar impact on the reproductive microbiome due to broad-spectrum bactericidal activity; therefore, there will not be significant differences amongst the microbiota after the completion of therapy.

**Methods:**

Cows from a central Pennsylvania dairy were screened for CM at 7 ± 3 days DIM using a Metricheck™ device (*n* = 351). Cows with red-brown watery discharge were diagnosed with CM and eligible for enrollment. Eligible cows (*n* = 77) were blocked by parity and randomly allocated to one of two treatments starting on the day of diagnosis: (1) Intrauterine dextrose (DEX, *n* = 38): 1 l of an intrauterine 50% dextrose solution for 3 days, and (2) Systemic ceftiofur (CONV, *n* = 39): two injections of ceftiofur (6.6 mg/Kg of BW; Excede, Zoetis Inc.) 72 h apart. Cows were evaluated for clinical cure rate at 7 ± 3 and 14 ± 3 days post-diagnosis. Vaginal discharge samples were collected using the Metricheck™ at enrollment day [study day (sd) 0, pre-treatment], sd 7, and sd 14 for a subset of enrolled cows (DEX = 13, CONV = 14). Vaginal discharge samples were analyzed with 16S rRNA sequencing to evaluate changes in the microbiome between treatments.

**Results:**

After treatment, there were only minor differences within the microbiome between the two treatments indicating the potential suitability of dextrose as an antibiotic-alternative treatment. Alpha diversity did not differ (Welch's *t*-test) between the treatments at any of the time points. Beta diversity based on PERMANOVA analysis did differ between treatments at sd 0 (*P* = 0.014) and again at sd 14 (*P* = 0.028), but not at sd 7 (*P* = 0.261).

**Discussion:**

While 16S rRNA analysis does not provide information on bacterial viability, the relative similarity of the microbiome between the two groups immediately following treatment might suggest that intrauterine dextrose could be utilized as an alternative treatment for CM.

## 1 Introduction

Metritis in dairy cattle is an ever-prevalent disease occurring in the first 21 days in milk (DIM) (1). Metritis is typically divided into two diseases: puerperal metritis, which includes evidence of systemic illness, such as fever, decreased milk yield, and lethargy, along with a fetid, red to brown watery uterine discharge as compared to clinical metritis, which presents with the same characteristics of discharge in the absence of systemic disease (1). Other interpretations of the clinical definition of metritis include having greater than 50% white purulent discharge and/or having sanguinopurulent mucus (2). A typical scoring system for metritis discharge collected by Metricheck™ device (Simcro Tech Ltd., Hamilton, New Zealand) includes grades of one through five, with one being characterized as normal discharge up to a grade five being described as fetid, red to brown watery discharge (3, 4). Cows with a score of four (red to brown watery discharge without fetid odor) and cows with a score of five (fetid, red to brown watery discharge) have a similar inflammatory response (5).

Metritis is most commonly reported to have about a 20% incidence rate amongst dairy herds, though this number can vary from 13 to 40% depending on location, parity, and season (2, 4, 6). With such a high disease incidence, there is a large interest in the economic impact of metritis on the dairy industry. The mean cost of a case of metritis amongst 16 different farms in the United States is $513 (4), which differs by whether treatment is administered (7). Ceftiofur is the antibiotic of choice for systemic treatment of metritis, with one study from Wisconsin showing that 68.6% of metritis cases were treated with the drug (8). Lack of treatment for metritis is seen as an animal welfare issue, as multiple studies conclude that metritis is associated with pain in cattle (9, 10). However, the use of antibiotics for metritis is not without cost. Antimicrobial resistance is a concerning trend throughout the healthcare fields, and studies are beginning to document the growing number of drug-resistant bacteria cultured from cases of metritis as well as from fecal samples of cows treated for metritis (11, 12). The need for antibiotic-alternative therapies has been identified in the dairy industry, and many alternatives have been suggested such as antibacterial peptides, pre and probiotics, phage therapy, and the development of novel vaccines (13, 14).

While culture-based studies remain a valuable source of information about the viability of metritis-associated bacteria and antimicrobial resistance patterns, culture-independent studies are changing how researchers think about the development and treatment of metritis in dairy cattle. Utilizing amplicon sequencing, Jeon et al. demonstrate that the uterine microbiome goes through rapid change between zero and six DIM with a significant increase in the relative abundance of *Bacteroides* in cattle developing metritis (15). A comparison of postpartum microbiomes finds that the vaginal and uterine microbiomes are most similar amongst specific operational taxonomic units and their relative abundances at seven DIM in cows who go on to develop endometritis compared to those cows that did not go on to develop endometritis (16). Ceftiofur treatment of metritis is correlated with decreased relative abundance of *Fusobacterium* at day 7 ± 1 day postpartum compared to untreated controls (3). As an alternative to standard antibiotic treatment, chitosan microparticles slow the progression of the uterine microbiota toward a healthy microbiome compared to cattle treated with ceftiofur (17). Overall, alternative treatment approaches for metritis should be evaluated for changes in the microbiome alongside clinical outcomes to fully understand the therapy's implications. The suggested alternative to antibiotic treatment is the use of dextrose to plasmolyze bacteria, thus causing bacterial death. The treatment approach was first described in the human literature as a therapy for wound healing (18). In another study measuring the water activity of 50% dextrose, the reported level for dextrose at room temperature is 0.93, with pure water being the reference value at 1.0. Substances with a water activity level of 0.85 or less are known to all bacterial growth due to the oncotic effects of the hyperosmolar environment causing cellular damage. That study also examined the ability of bacterial pathogens to grow in a 50% dextrose solution and only saw bacterial growth in the vials of dextrose stored under refrigerated conditions but not in those under room temperature. These results indicate that a 50% dextrose solution will inhibit some but not all bacterial growth due to its lower water activity level (19). In cattle, there have been mixed results when treating the reproductive tract with dextrose. Treatment with intrauterine dextrose for clinical endometritis (CE) in dairy cattle resulted in better clinical cure rates compared to control cows 14 days post-treatment and a similar clinical cure proportion as ceftiofur (20). However, a further study of intrauterine dextrose as a treatment for CE had a statistical tendency to decrease cure rate (21). Another study examining the benefits of intrauterine dextrose for treating cows with purulent vaginal discharge saw an increase in pregnancy per artificial insemination rate for intrauterine dextrose-treated cows as compared to control (22). Despite the conflicting studies on intrauterine dextrose's benefit in treating clinical endometritis, the use of intrauterine dextrose as a preventative therapy for metritis has only recently been evaluated without investigating its impact on the reproductive microbiome (23, 24). Therefore, this study aimed to assess microbial changes within the vaginal microbiome of cows treated with intrauterine dextrose as compared to ceftiofur. We hypothesize that both treatment methods will have a similar impact on the reproductive microbiome due to broad-spectrum bactericidal activity and prior data indicating similar in vitro bactericidal properties of a sugar solution and ceftiofur (18, 25), and therefore, there will not be significant differences amongst the microbiota after the completion of therapy.

## 2 Methods

### 2.1 Ethics statement

This study was approved by The Pennsylvania State University Institutional Animal Care and Use Committee (Protocol # 201900854).

### 2.2 Animals and management

The study was performed on a 700-milking cow dairy in central Pennsylvania (26). The cows were milked three times a day, and the yearly rolling herd average milk yield was 11,143 kg. Around 14 (±3) days before expected calving date, cows and heifers were housed together in a pen with deep straw bedding and checked every one to three hours for signs of labor onset - at which time they were moved to a small, straw-bedded calving pen for calving through to collection of colostrum. Post-calving cows and heifers were then moved to a postpartum pen for four to five days before finishing their lactation in naturally ventilated free-stall pens with deep sand bedding equipped with additional fans and sprinklers. The cows were fed a TMR diet twice daily that met or exceeded dietary nutritional requirements (27).

### 2.3 Enrollment, treatment, and sample collection

Eligible animals were enrolled in the study from May 2019 until August 2020. Cows at 7 ± 3 DIM, a typical timepoint for cows developing metritis, were screened for inclusion in the study if they had no recorded previous or current non-reproductive health events from the time of calving, such as lameness; however, cows that had dystocia, stillbirth, twins, or retained fetal membranes were included in the study as these diseases often precede the development of metritis. Overall, 351 Holstein cows were screened by Metricheck™ device (Simcro Tech Ltd., Hamilton, New Zealand) based on a five-point scoring system for vaginal discharge (i.e., one- clear fluid, two- < 50% white purulent fluid, three- >50% white purulent fluid, four- red-brownish fluid without fetid smell, and five- fetid red-brownish watery fluid) (5). A vaginal discharge score of > 4 meant the cow was diagnosed with clinical metritis (5). Cows diagnosed with clinical metritis were then enrolled into the treatment arm of the study.

**Figure 1:**
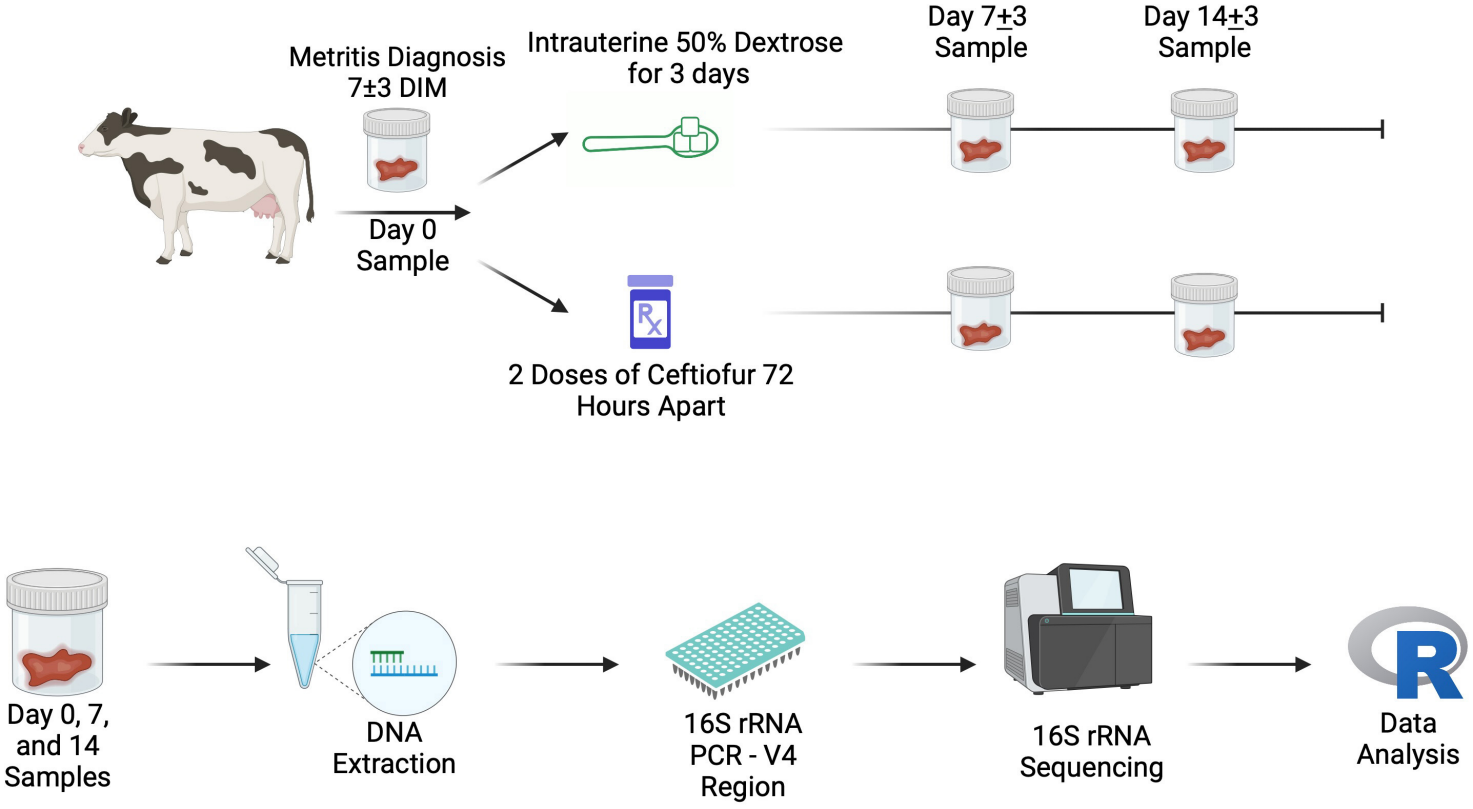
On-farm sampling outline **(Top)** and laboratory workflow schematic **(Bottom)**. Created in BioRender. Lection, J. (2024) https://BioRender.com/y71j645. DIM, days in milk.

After enrollment, cows were blocked by parity and randomized to one of two treatment groups: 1. Intrauterine Dextrose (**DEX**, *n* = 36): starting on study day 0 (7 ± 3 DIM), cows received an intrauterine infusion of 1L of 50% dextrose solution (AgriLabs) every 24 h for three days or 2. Systemic Antibiotics (**CONV**, *n* = 38): Two subcutaneous injections of ceftiofur crystalline free acid (6.6 mg/Kg of BW; Excede, Zoetis Inc.) 72 h apart. In the cows receiving the DEX treatment, as much abnormal fluid was removed from the uterus as possible using a stainless-steel half-inch infusion rod and transrectal uterine massage. Both the research team and the farm herd manager, who was trained before the study, administered treatments to enrolled cows. On study day 0, body condition score of the enrolled subjects was assessed on a 5-point scale (28). Samples of vaginal discharge were collected at study day 0 (7 ± 3 DIM) before treatment, study day seven (14 ± 3 DIM), and study day 14 (21 ± 3 DIM) and saved in conical vials, stored at −80°C until DNA extraction (Figure 1). On study day seven and study day 14, the research team assessed each cow for clinical cure of metritis (vaginal discharge score < 3). A random subset of the vaginal discharge samples was chosen for microbial community analysis based on a previously published study examining the impact of antibiotic treatment on the metritis microbiome that used group sizes of 13–15 samples per treatment group (29).

### 2.4 DNA extraction, 16S rRNA PCR, and amplicon sequencing

A random subset of vaginal samples was selected for inclusion in this microbiome study (DEX *n* = 13, CONV *n* = 14). Vaginal discharge samples were thawed, and DNA was extracted with the MagMAX™ CORE Nucleic Acid Purification Kit according to the manufacturer's instructions (ThermoFisher Scientific, Waltham, MA, USA). A known microbial community sample was used as a positive control (ZymoBIOMICS Microbial Community Standard, Zymo Research, Irvine, CA, USA) was included to ensure successful extraction and a negative control (Nuclease Free Water, VWR, Radnor, PA, USA) was extracted to be able to check for contamination. Extracted DNA concentration and quality were checked for each sample using the Nanodrop One (Thermo Fisher Scientific, Waltham, MA). The V4 hypervariable region of the 16S rRNA gene was amplified using the 515 forward primer (5′TCGTCGGCAGCGTCAGATGTGTATAAGAGACAGGTGYCAGCMGCCGCGGTAA3′) and 806 reverse primer, (5′GTCTCGTGGGCTCGGAGATGTGTATAAGAGACAGGGACTACNVGGGTWTCTAAT 3′) according to the Earth Microbiome Project specifications (30–32). A sample of known microbial community composition (ZymoBIOMICS Microbial Community DNA Standard, Zymo Research, Irvine, CA, USA) was used to determine the successful amplification of the 16S rRNA gene. The PCR conditions were 94°C for 3 min, 25 cycles of 94°C for 45 s, 50°C for 60 s, and 72°C for 90 s, then 72°C for 10 min, and held at 4°C. PCR product amplification was checked for each sample on a 2% agarose gel. Samples were sent to Novogene Corporation Inc. (Durham, NC) for amplicon sequencing on the NovaSeq platform (Illumina, Inc., San Diego, CA).

### 2.5 Bioinformatics

Sequencing read quality was determined with FastQC (v. 0.11.9), and reads were trimmed with Trimmomatic (v. 0.39) with a minimum read length of 100 base pairs and a minimum Phred score of 20 (33, 34). Reads were processed through the dada2 pipeline to remove chimeras, generate amplicon sequence variants (ASVs) and merge paired-end reads as implemented in the dada2 R package (35). Taxonomy was assigned via the Silva database (v.138.1) (36). The decontam package (v. 0.1.10) was used to remove reads that were the result of contamination based on the negative DNA extraction and PCR controls (37). Rare taxa with total mean relative abundance of less than 0.00001 were also removed. The phyloseq package (v. 1.42.0) was used to calculate alpha (Shannon's index) and beta diversity (Aitchison distances) (38). Statistical analyses of alpha diversity were performed with a linear model with treatment as an independent variable and tested with Welch's t-test (39). A Shapiro-Wilk test was used to assess the data's normality before using a t-test to detect significant differences (40). Beta dispersion (homogeneity of variance) was tested, followed by beta diversity (the model included treatment as the sole independent variable was tested with PERMANOVA of Atchison's distance with 9,999 permutations) using the adonis function of the vegan package (v. 2.6-4) (41). For differential relative abundance, the package ALDEx2 (v. 1.30.0) was used with 2000 Monte Carlo instances and an inter-quartile log-ratio approach to account for asymmetry in the data set and then tested with a t-test along with the Benjamini-Hochberg (BH) procedure for post hoc testing (42–44). Based on the findings of McGovern et al., gene set enrichment analysis was carried out using the effect size output from ALDEx2 and the package fsgea (v. 1.24.0) to determine whether there was significant enrichment of metritis-associated bacteria in either treatment group at any of the three time points (45). The analysis was performed twice, once labeling the metritis-associated bacteria to contain bacteria in the genera *Escherichia, Trueperella, Fusobacterium*, and *Prevotella* to represent metritis-associated bacteria found in culture-based studies and a second time with the genera *Bacteroides, Porphyromonas*, and *Fusobacterium* to represent bacteria found in uterine dysbiosis from metagenomic studies (46). The gene set enrichment analysis was repeated including an error term of −0.5 to 0.5 in increments of 0.1 to account for errors in assumptions about the total number of bacteria inhabiting the vagina, to help ensure that the results would hold whether there were in actuality more or fewer bacteria than assumed (45). Statistical differences were set at a significance level of *P* < 0.05 with analyses conducted with R (v. 4.2.2).

## 3 Results

### 3.1 Study population and clinical results

The random subset of discharge samples included in the microbiome analysis contained 13 cows treated with intrauterine dextrose (DEX) and 14 cows treated with systemic antibiotics (CONV). Of the cows treated with intrauterine dextrose, nine were multiparous (average lactation = 3.56 ± 1.51), and four were primiparous. Of the cows treated with systemic antibiotics, 12 were multiparous (average lactation = 3.5 ± 1), and two were primiparous. The clinical cure rate at study day seven was 76.92% (10/13) for DEX and 71.43% (10/14) for CONV, and for study day 14, DEX was 11/13 (84.62%) and CONV was 13/14 (92.86%).

### 3.2 Taxonomy

**Figure 2:**
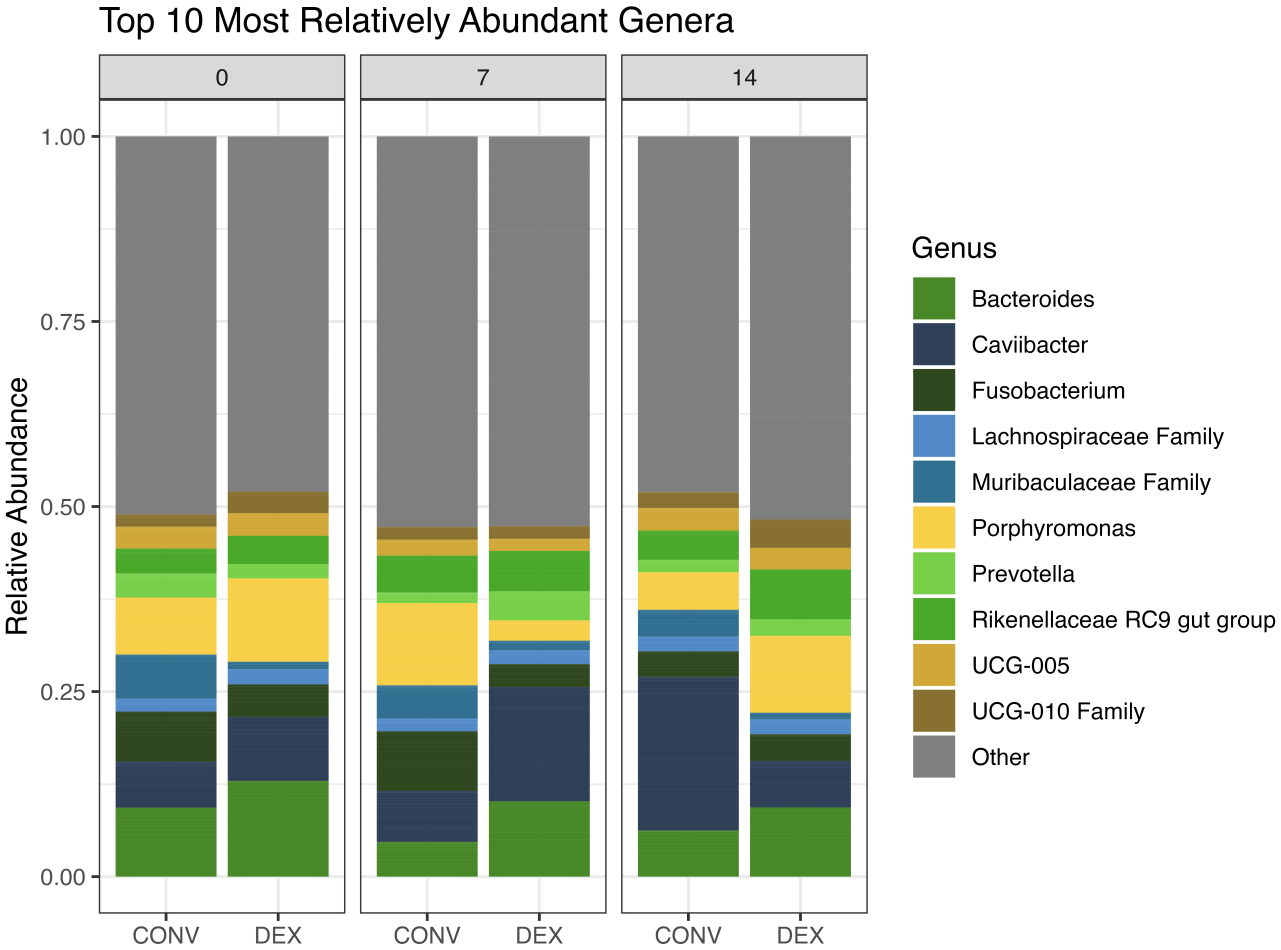
The top 10 relatively abundant genera for each treatment group at each sampling time point. Other represents the rest of the genera not included in the top 10.

In the 81 total discharge samples and three technical controls, the average number of reads returned per sample was 125,972, with a range of 49,972 to 148,110 for a total of 10,581,636 reads. After filtering and removal of controls, 1,952 taxa (ASV) remained, comprising 374 genera. The top 10 genera across both treatment groups are presented in Figure 2. Overall, the most relatively abundant three genera were *Caviibacter* (10.6% relative abundance of all genera), *Bacteroides* (8.65%), and *Porphyromonas* (7.48%). At study day 0, the top three most abundant genera were *Bacteroides* (12.2%), *Porphyromonas* (7.59%), and *Fusobacterium* (7.49%). The abundant genera findings for cows treated with systemic antibiotics vs. intrauterine dextrose are shown in Table 1.

**Table 1:** Top three highest relatively abundant genera by treatment at each sampling point, including each genera's percentage of relative abundance.

**Study day 0**			**Study day 7**			**Study day 14**		
**Dextrose**								
1	*Bacteroides*	14.50%	1	*Caviibacter*	15.20%	1	*Porphyromonas*	9.72%
2	*Porphyromonas*	7.40%	2	*Bacteroides*	9.29%	2	*Bacteroides*	8.20%
3	*Fusobacterium*	7.40%	3	*Rikenellaceae RC9 gut group*	4.19%	3	*Rikenellaceae RC9 gut group*	6.31%
			4	*Fusobacterium*	3.89%	5	*Fusobacterium*	3.82%
**Antibiotics**								
1	*Bacteroides*	10.10%	1	*Porphyromonas*	11.40%	1	*Caviibacter*	20.20%
2	*Porphyromonas*	7.70%	2	*Fusobacterium*	8.33%	2	*Bacteroides*	5.73%
3	*Caviibacter*	7.75%	3	*Caviibacter*	7.40%	3	*Porphyromonas*	5.25%
4	*Fusobacterium*	7.58%				5	*Fusobacterium*	3.90%

If *Fusobacterium* is not in the top 3, its position in the genera rankings is noted along with its relative abundance percentage.

### 3.3 Diversity indices

**Figure 3:**
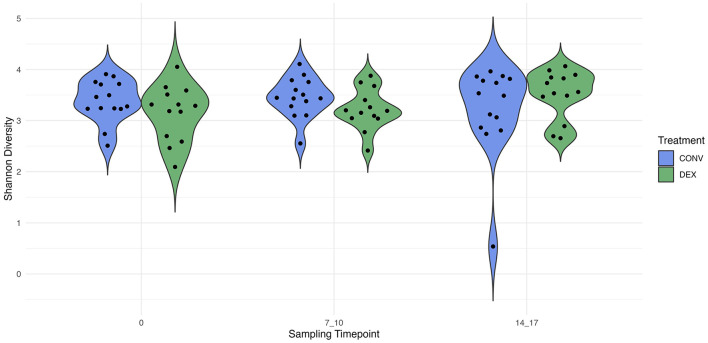
The distribution of alpha diversity, measured with Shannon's index, for each treatment group at each sampling timepoint. The estimate of Shannon's diversity index is illustrated on the y-axis.

**Figure 4:**
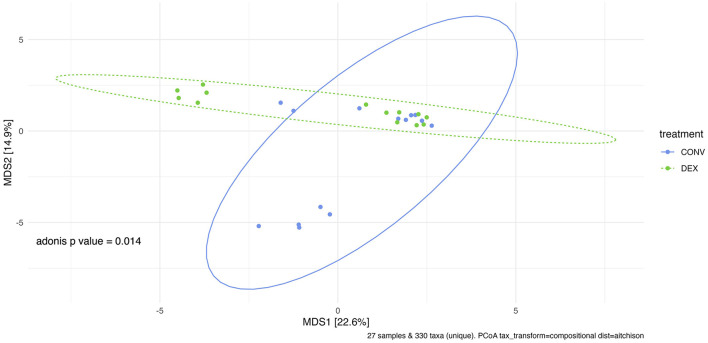
Principal coordinate analysis of results from PERMANOVA estimating beta diversity between treatment groups at sampling time point 0 with Adonis *P*-value of 0.014. There is a significant difference between the treatment groups' community structure prior to any treatment administration.

**Figure 5:**
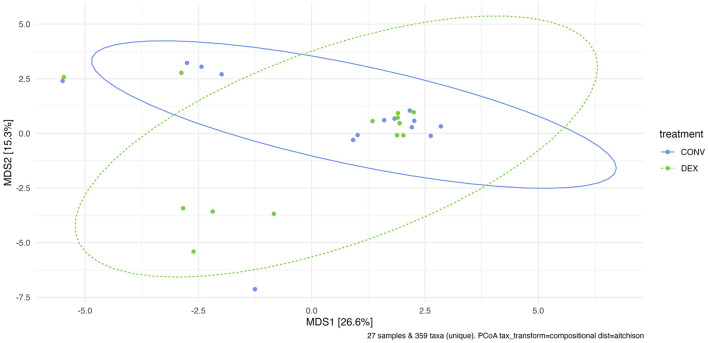
Principal coordinate analysis of results from PERMANOVA estimating beta diversity between treatment groups at sampling time point 7. Adonis *P*-value is 0.261—there is not a significantly different community structure at the time point directly after treatment was finished.

**Figure 6:**
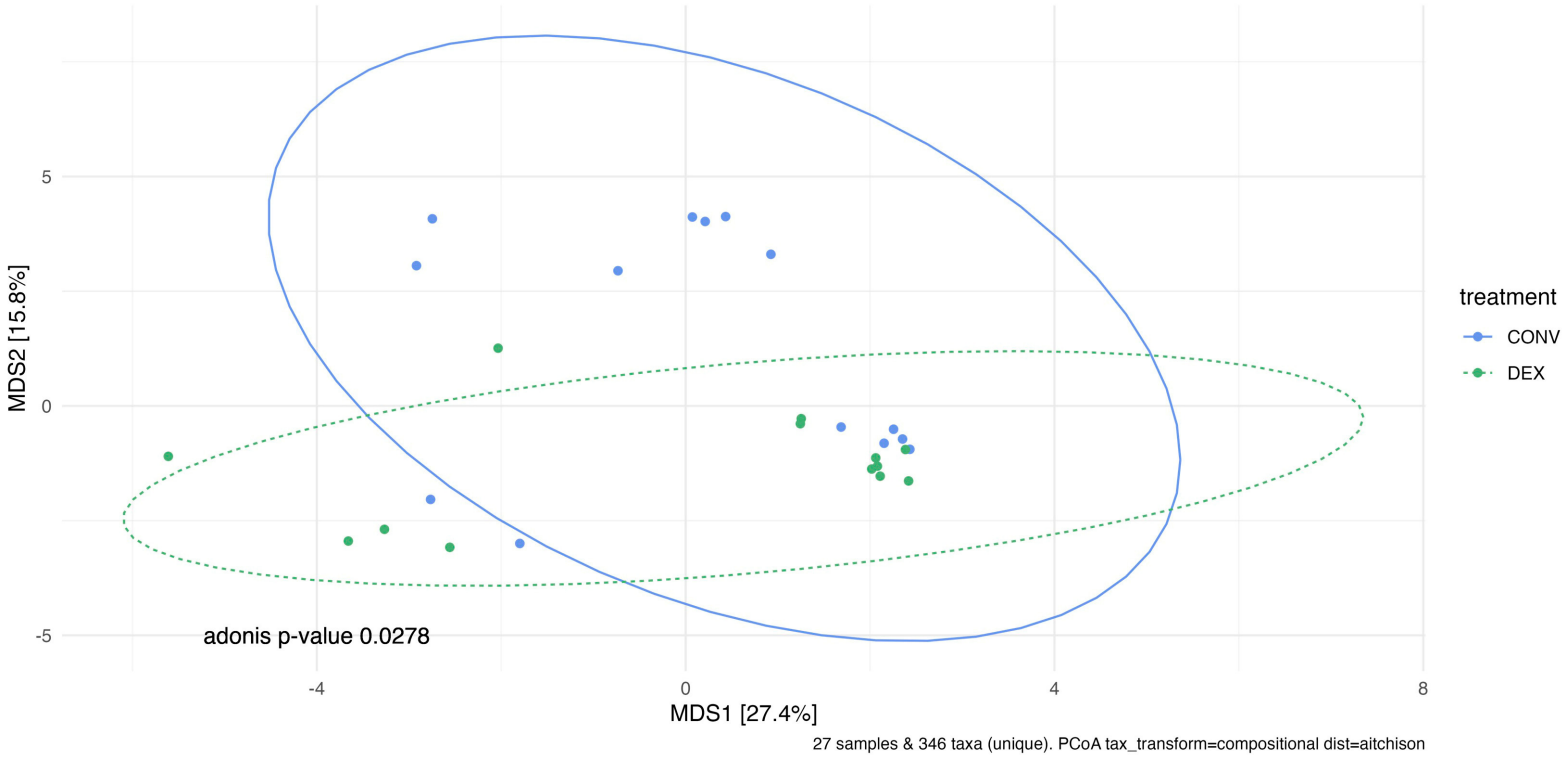
Principal coordinate analysis of results from PERMANOVA estimating beta diversity between treatment groups at sampling time point 14. Adonis *P*-value is 0.0278—there is a significantly different community structure at the final sampling time point.

Shannon's diversity index was used to assess alpha diversity between treatment groups at each point in time. There were no differences between CONV and DEX at day zero, day seven, or day 14 (Table 2, Figure 3). Beta dispersion did not significantly differ at any sampling timepoint (*P* > 0.05). For beta-diversity, a permutational ANOVA was performed on Aitchison's distances and yielded a significant difference between treatments at study day 0 (pseudo-F: 2.06, *P* = 0.01, Figure 4), but not at study day seven (pseudo-F: 1.15, *P* = 0.26, Figure 5), though a significant difference was found at study day 14 as well (pseudo-F: 2.07, *P* = 0.03, Figure 6). Beta diversity did not differ by parity at baseline (pseudo-F: 0.92, *P* = 0.51).

**Table 2:** Mean estimates and standard deviation for Shannon's Diversity Index per sampling timepoint and treatment group.

	**Intrauterine dextrose**	**Systemic antibiotics**
Study day 0	3.15 ± 0.55	3.38 ± 0.41
Study day 7	3.22 ± 0.40	3.46 ± 0.39
Study day 14	3.51 ± 0.48	3.23 ± 0.89

### 3.4 Differential relative abundance

Differential relative abundance testing was performed between treatment groups at each of the three study time points as well as between the treatment groups across the entire study. After correcting for multiple comparisons, no taxa had significant differential relative abundance between treatment groups at any of the individual time points. When all three time points were grouped together, one taxon was significantly more relatively abundant after post hoc testing in the CONV group: the genus *Peptococcus*, which is in the Firmicutes phylum (effect size: −0.689, Adj. *P* = 0.02).

### 3.5 Gene set enrichment analysis

Bacterial genera were assigned as either metritis-associated or no association with metritis based on culture-based studies for the first round of gene set enrichment analysis, and for the second round of analysis, genera were assigned based on metagenomic studies. Gene set analysis was used to determine whether there was significant enrichment of metritis-associated genera in either of the treatment groups at any of the three time points; however, neither of the analyses showed significant enrichment (*P* > 0.05).

## 4 Discussion

We have evaluated the effects of intrauterine dextrose as an experimental nonantibiotic treatment for clinical metritis on the reproductive microbiome in dairy cattle compared to systemic antibiotic treatment. To the best of our knowledge, the impact of intrauterine dextrose on the reproductive microbiome of cows with clinical metritis has not been previously studied. Still, it warrants consideration when suggesting a novel treatment protocol as to ensure the treatment does not cause a dysbiosis of the reproductive microbiome. Our results show very few differences in the community richness, evenness, and structure between CONV and DEX treatments. Metritis-associated bacteria were not significantly enriched in either of the treatment groups, thus providing evidence that intrauterine dextrose may be a viable alternative treatment for clinical metritis to systemic antibiotics.

At study day zero, the top three most abundant genera in the present study were *Bacteroides, Porphyromonas*, and *Fusobacterium*, which matches the findings of Jeon et al. on day 6 ± 2 days postpartum, even though their definition of metritis only included the score of five (15). The results of that study may closely compare to the current study due to both using the V4 hypervariable region of the 16S rRNA gene. Since the microbial communities were dominated by similar genera across metritis scores of four and five, these findings support the results of Barragan et al. that a score of four or a score of five both represent a diseased postpartum uterus (5). In the study by Jeon et al., animals were sampled by a guarded swab into the uterus instead of sampling vaginal discharge, which supports that early in the postpartum period, there may be a shared microbial community between both the uterus and vagina, as shown in Miranda-CasoLuengo et al. (16). A third study found the most relatively abundant three genera were *Bacteroides, Porphyromonas*, and *Fusobacterium* from endometrial swabs taken at 5-10 DIM with pyrosequencing of the V1-V3 hypervariable region, and shotgun sequencing of cows between 3 and 12 DIM revealed the same top three genera in cattle with metritis after sampling the uterus with a guarded swab (47). This supports the view of a core microbial community in metritic cattle reproductive microbiomes in the first 12 days postpartum that can potentially be used for early screening of metritis in cattle prior to changes in vaginal discharge.

*Fusobacterium* is the one genera that overlaps between metritis-associated pathogens in culture-based and culture-independent studies and is decreased by ceftiofur treatment (3). Interestingly, there was no significant difference in the differential relative abundance of *Fusobacterium* between treatments at any of the three time points in the present study, even though the relative abundance of *Fusobacterium* for dextrose-treated cattle decreased from 7.4% before treatment to 3.89% on study day seven and 3.82% on study day 14. In comparison, for ceftiofur-treated cows, the relative abundance of *Fusobacterium* on study day zero was 7.58%; on study day seven, it was 8.33%, but by study day 14, it was 3.90%. Since these results are not statistically significant, it appears that there is no clinical difference in the ability of intrauterine dextrose to treat *Fusobacterium* as compared to the antibiotic treatment. Gene set enrichment analysis did not show any significant enrichment of metritis-associated bacteria in one treatment vs. the other at any of the three time points. The analysis was rigorously carried out by adding an error term to the model to allow for incorrect assumptions in the total number of bacteria in the reproductive tract; however, the conclusion still held that metritis-associated bacteria, taken as a group, were not significantly more present in either treatment group.

While *Fusobacterium* did not differ based on differential relative abundance, *Peptococcus* was increased in the systemic antibiotic group. It is in the *Peptococcaceae* family and has been previously described as part of the healthy cow vaginal microbiome although it may be linked to other inflammatory diseases in cattle, such as pneumonia, which is in contrast to the genus being found in healthy cattle reproductive tracts (48). With so few reports of *Peptococcus* in the cattle reproductive literature, it is difficult to determine whether its increased differential relative abundance in cows treated with antibiotics plays a defining role in treatment success as compared to the alternative treatment.

A recent metagenomic study of the postpartum uterine microbiome saw significant differences in Shannon's diversity between control cows and those developing metritis, which indicates a dysbiosis in the metritic uterus (49). The present study showed no difference in alpha diversity before treatment, showing a homogenous group of enrolled cattle, and no difference in alpha diversity at either time point after treatment showing that neither treatment created a further dysbiosis in the uterus. If one treatment harmed the reproductive microbiome, there would likely be differences in alpha diversity, as was seen between healthy and metritic cattle in the aforementioned study. However, the two treatments differed in beta diversity on study day 0. Further PERMANOVAs were carried out based on the available metadata to try to explain the difference in groups, but there were no significant findings. Based on the ordination in Figure 4, many of the cows cluster together between the two treatment groups, so the outlying cows are likely driving the significant finding. The study was designed to take into account biologically significant factors that may affect the bacterial composition of the reproductive tract including parity as well as using cows that were all similarly managed on the same farm; however, there can be other intrinsic factors to the cows such as immunity status or stage of the disease process that could explain this statistical difference. Despite the difference at study day 0, the first timepoint after treatment showed no significant difference in beta diversity. Ceftiofur has previously been shown to shift the beta diversity of the microbiome of dairy cattle to increased homogeneity, so it is possible in the current study that both treatments similarly altered the reproductive microbiome (3). The difference in beta diversity reappeared on study day 14, but changes in the reproductive microbiome related to metritis are challenging to interpret at this time point, as this sampling was around 21 days postpartum. Day 21 postpartum is traditionally the beginning of the time period for diagnosis of endometritis, so the changes may be related to the development of that disease (1).

A limitation of the present study is the lack of an untreated group of cows. Since not providing treatment to cows with metritis can be considered a welfare issue due to the systemic disease that can result from the infection as well as elevated markers of pain associated with the disease, the authors did not elect to have a group of metritic cattle go without treatment (1, 10). One alternative could have been to sample cows without metritis or other postpartum disease at study time points zero, seven, and 14 days. With those samples, the impact of metritis on the reproductive microbiome could be established at timepoint 0, and then after treatments, the changes in the microbiome by treatment group could be compared to a healthy microbiome at that study time point. However, sampling healthy cows at these time points is not a perfect alternative for a no-treatment group, and therefore, the results of this study should be interpreted as other clinical trials—a novel treatment vs. a gold standard treatment, rather than showing the impact of a novel treatment on the disease process itself. It is known from previous studies that the risk for metritis self-cure is significantly lower than the risk for cure with ceftiofur treatment, and therefore, it was elected to forego the untreated metritis group as it does not address the question of how the impact of dextrose on the reproductive microbiome differs from the impact of ceftiofur.

A second limitation of this study is sampling the discharge from the vagina of the cow compared to the uterus as a less-invasive proxy of the microbiome. Prior research examined the similarity of the uterine and vaginal microbiomes in postpartum cows, which went on to develop clinical endometritis, and found these two microbiomes were most similar at day seven postpartum as compared to cows that remained healthy (16). Since in the current study, the impact of the treatment was assessed around days 14 (study day seven) and 21 (study day 14) postpartum, potentially, the vaginal microbiome is not as similar to the uterine microbiome as at study day 0. Bacteriological studies of the postpartum uterus and vagina also showed a significant correlation between possible metritis-causing bacteria in the vagina and uterus (50). Based on that evidence along with the convenience of the Metricheck™ device as well as the clinical diagnosis of metritis being based on the sampling of vaginal discharge, the authors wanted to carry out this study by taking samples in a clinically applicable manner that can be applied to future studies. One final limitation of the study design is the lack of blinding in treatment administration to each cow due to the difference in route of administration—injection compared to intrauterine infusion. Despite the lack of blinding in that aspect of the study, the treatment outcome assessment was blinded as the two researchers who performed the vaginal discharge scoring were not aware of which cow received which treatment, thus preventing bias in the results.

An intriguing future direction for this study would be to follow the cows past the voluntary waiting period, typically assumed to be around 60 DIM (51). Following the microbiome until this point would provide information about whether there were any longer-term consequences of either treatment method. This future direction would also demonstrate the microbial population around the time of first insemination and the potential correlation between changes in the reproductive microbiome and fertility outcomes.

In conclusion, this study examined the impact of two different treatment protocols for clinical metritis: a standard systemic antibiotic course of therapy as well as an antibiotic-alternative—intrauterine dextrose. Vaginal bacterial richness and evenness did not significantly differ between the two groups at any of the study time points; however, bacterial community structure was significantly different before, but not after, treatment. In the most critical time point, study day seven, the community structure showed no significant differences, indicating a similar impact of treatment on the reproductive microbiome regardless of treatment protocol. There were minimal differences in differential relative abundance of bacterial genera between treatments, and gene set analysis failed to show significant enrichment of metritis-associated bacteria between the two treatments at any of the time points. The clinical results from the broader study of utilizing intrauterine dextrose for metritis indicate that intrauterine dextrose may be a viable treatment option for cows with a vaginal discharge score of four as well as can be an alternative metritis treatment option in situations where antibiotics cannot be utilized such as the organic dairy industry (26). The need for antibiotic-alternatives have been well documented in the dairy industry, with modulation of the microbiome commonly being discussed as a potential alternative treatment (13, 14). The results of that study along with the conclusion of the current study that impact of intrauterine dextrose on the reproductive microbiome compared to treating with antibiotics appears to be minimal, further suggesting it may be a viable alternative therapy to minimize the overuse of antimicrobials.

## Data Availability

The datasets presented in this study can be found in online repositories. The names of the repository/repositories and accession number(s) can be found below: https://www.ncbi.nlm.nih.gov/bioproject/PRJNA1128599.

## References

[1] Sheldon IM Lewis GS LeBlanc S Gilbert RO. Defining postpartum uterine disease in cattle. Theriogenology. (2006) 65:1516–30. 10.1016/j.theriogenology.2005.08.021 16226305

[2] Molinari PCC Dahl GE Sheldon IM Bromfield JJ. Effect of calving season on metritis incidence and bacterial content of the vagina in dairy cows. Theriogenology. (2022) 191:67–76. 10.1016/j.theriogenology.2022.08.001 35970030

[3] Jeon SJ Cunha F Daetz R Bicalho RC Lima S Galvão KN. Ceftiofur reduced Fusobacterium leading to uterine microbiota alteration in dairy cows with metritis. Anim Microbiome. (2021) 3:15. 10.1186/s42523-021-00077-5 33509303 PMC7844903

[4] Pérez-Báez J Silva TV Risco CA Chebel RC Cunha F De Vries A. The economic cost of metritis in dairy herds. J Dairy Sci. (2021) 104:3158–68. 10.3168/jds.2020-19125 33455790

[5] Barragan AA Lakritz J Carman MK Bas S Hovingh E Schuenemann GM. Short communication: assessment of biomarkers of inflammation in the vaginal discharge of postpartum dairy cows diagnosed with clinical metritis. J Dairy Sci. (2019) 102:7469–75. 10.3168/jds.2018-15854 31202654

[6] Sheldon IM Williams EJ Miller ANA Nash DM Herath S. Uterine diseases in cattle after parturition. Vet J. (2008) 176:115. 10.1016/j.tvjl.2007.12.031 18329302 PMC2706386

[7] Lima FS Vieira-Neto A Snodgrass JA De Vries A Santos JEP. Economic comparison of systemic antimicrobial therapies for metritis in dairy cows. J Dairy Sci. (2019) 102:7345–58. 10.3168/jds.2018-15383 31178192

[8] Gonçalves JL de Campos JL Steinberger AJ Safdar N Kates A Sethi A. Incidence and Treatments of bovine mastitis and other diseases on 37 dairy farms in Wisconsin. Pathogens. (2022) 11:1282. 10.3390/pathogens11111282 36365033 PMC9698317

[9] Stojkov J von Keyserlingk MAG Marchant-Forde JN Weary DM. Assessment of visceral pain associated with metritis in dairy cows. J Dairy Sci. (2015) 98:5352–61. 10.3168/jds.2014-9296 26074240

[10] Barragan AA Piñeiro JM Schuenemann GM Rajala-Schultz PJ Sanders DE Lakritz J. Assessment of daily activity patterns and biomarkers of pain, inflammation, and stress in lactating dairy cows diagnosed with clinical metritis. J Dairy Sci. (2018) 101:8248–58. 10.3168/jds.2018-14510 29937269

[11] Taylor EA Jordan ER Garcia JA Hagevoort GR Norman KN Lawhon SD. Effects of two-dose ceftiofur treatment for metritis on the temporal dynamics of antimicrobial resistance among fecal *Escherichia coli* in Holstein-Friesian dairy cows. PLoS ONE. (2019) 14:e0220068. 10.1371/journal.pone.0220068 31329639 PMC6645674

[12] Basbas C Garzon A Silva-Del-Rio N Byrne BA Karle B Aly SS. Evaluation of antimicrobial resistance and risk factors for recovery of intrauterine *Escherichia coli* from cows with metritis on California commercial dairy farms. Sci Rep. (2022) 12:13937. 10.1038/s41598-022-18347-w 35978077 PMC9386028

[13] Sharma C Rokana N Chandra M Singh BP Gulhane RD Gill JPS. Antimicrobial resistance: its surveillance, impact, and alternative management strategies in dairy animals. Front Vet Sci. (2017) 4:237. 10.3389/fvets.2017.00237 29359135 PMC5766636

[14] Trevisi E Zecconi A Cogrossi S Razzuoli E Grossi P Amadori M. Strategies for reduced antibiotic usage in dairy cattle farms. Res Vet Sci. (2014) 96:229–33. 10.1016/j.rvsc.2014.01.001 24508188

[15] Jeon SJ Vieira-Neto A Gobikrushanth M Daetz R Mingoti RD Parize ACB. Uterine microbiota progression from calving until establishment of metritis in dairy cows. Appl Environ Microbiol. (2015) 81:6324. 10.1128/AEM.01753-15 26150453 PMC4542247

[16] Miranda-CasoLuengo R Lu J Williams EJ Miranda-CasoLuengo AA Carrington SD O Evans AC. Delayed differentiation of vaginal and uterine microbiomes in dairy cows developing postpartum endometritis. PLoS ONE. (2019) 14:e0200974. 10.1371/journal.pone.0200974 30629579 PMC6328119

[17] Galvão KN de Oliveira EB Cunha F Daetz R Jones K Ma Z. Effect of chitosan microparticles on the uterine microbiome of dairy cows with metritis. Appl Environ Microbiol. (2020) 86:20. 10.1128/AEM.01066-20 32651210 PMC7480369

[18] Chirife J Herszage L Joseph A Kohn ES. In vitro study of bacterial growth inhibition in concentrated sugar solutions: microbiological basis for the use of sugar in treating infected wounds. Antimicrob Agents Chemother. (1983) 23:766. 10.1128/AAC.23.5.766 6870223 PMC184812

[19] Marshall KA Brooks AC Hammac GK Thomovsky EJ Johnson PA. Prevalence of bacterial contamination in 50% dextrose vials in varying storage conditions after multiple punctures: bacterial contamination in 50% dextrose vials. J Small Anim Pract. (2018) 59:758–62. 10.1111/jsap.12906 29972244

[20] Brick TA Schuenemann GM Bas S Daniels JB Pinto CR Rings DM. Effect of intrauterine dextrose or antibiotic therapy on reproductive performance of lactating dairy cows diagnosed with clinical endometritis. J Dairy Sci. (2012) 95:1894–905. 10.3168/jds.2011-4892 22459836

[21] Machado VS Oikonomou G Ganda EK Stephens L Milhomem M Freitas GL. The effect of intrauterine infusion of dextrose on clinical endometritis cure rate and reproductive performance of dairy cows. J Dairy Sci. (2015) 98:3849–58. 10.3168/jds.2014-9046 25795484

[22] Maquivar MG Barragan AA Velez JS Bothe H Schuenemann GM. Effect of intrauterine dextrose on reproductive performance of lactating dairy cows diagnosed with purulent vaginal discharge under certified organic management. J Dairy Sci. (2015) 98:3876–86. 10.3168/jds.2014-9081 25828665

[23] Mattice H Jimenez E Hovingh E Bas S Martinez M Barragan AA. Postpartum intrauterine dextrose infusion: Effects on uterine health, metabolic stress, systemic inflammation, and daily milk yield in clinically healthy dairy cows. JDS Commun. (2023) 4:121–6. 10.3168/jdsc.2022-0310 36974215 PMC10039235

[24] Maquivar MG Barragan AA Velez JS Bothe H Schuenemann GM. Effect of intrauterine dextrose or iodine infusions on clinical cure and reproductive performance of lactating dairy cows with clinical metritis under certified organic management. 2013 JOINT ANNUAL MEETING ABSTRACTS. J Dairy Sci. (2013). p.382. 10.3168/jds.2014-9081 25828665

[25] Salmon SA Watts JL Yancey RJ Jr. In vitro activity of ceftiofur and its primary metabolite, desfuroylceftiofur, against organisms of veterinary importance. J Vet Diagn Invest. (1996) 8:332–6. 10.1177/104063879600800309 8844576

[26] Hamilton J Jimenez E Zarei P Lection J Sorto R Hovingh E. Exploring vaginal discharge scoring to assess clinical metritis severity: comparison between intrauterine dextrose and systemic antibiotics treatments. Vet J. (2024) 304:106103. 10.1016/j.tvjl.2024.106103 38522779

[27] National National Academies of Sciences Engineering Medicine. Nutrient Requirements of Dairy Cattle. Washington, DC: National Academy Press (2021). 482 p. 38386771

[28] Ferguson JD Galligan DT Thomsen N. Principal descriptors of body condition score in Holstein cows. J Dairy Sci. (1994) 77:2695–703. 10.3168/jds.S0022-0302(94)77212-X 7814740

[29] Jeon SJ Lima FS Vieira-Neto A Machado VS Lima SF Bicalho RC. Shift of uterine microbiota associated with antibiotic treatment and cure of metritis in dairy cows. Vet Microbiol. (2017) 214:132–9. 10.1016/j.vetmic.2017.12.022 29408025

[30] Parada AE Needham DM Fuhrman JA. Every base matters: assessing small subunit rRNA primers for marine microbiomes with mock communities, time series and global field samples. Environ Microbiol. (2015) 18:1403–14. 10.1111/1462-2920.13023 26271760

[31] Apprill A Mcnally S Parsons R Weber L. Minor revision to V4 region SSU rRNA 806R gene primer greatly increases detection of SAR11 bacterioplankton. Aquat Microb Ecol. (2015) 75:129–37. 10.3354/ame01753

[32] Thompson L Sanders JG McDonald D Amir A Ladau J Locey KJ. A communal catalogue reveals Earth's multiscale microbial diversity. Nature. (2017) 551:457–63. 29088705 10.1038/nature24621 PMC6192678

[33] Andrews S. FastQC: A Quality Control Tool for High Throughput Sequence Data (2010). Available at: http://www.bioinformatics.babraham.ac.uk/projects/fastqc

[34] Bolger AM Lohse M Usadel B. Genome analysis trimmomatic: a flexible trimmer for Illumina sequence data. Bioinformatics. (2014) 30:2114–20. 10.1093/bioinformatics/btu170 24695404 PMC4103590

[35] Callahan BJ McMurdie PJ Rosen MJ Han AW Johnson AJA Holmes SP. DADA2: High resolution sample inference from Illumina amplicon data. Nat Methods. (2016) 13:581. 10.1038/nmeth.3869 27214047 PMC4927377

[36] Quast C Pruesse E Yilmaz P Gerken J Schweer T Yarza P. The SILVA ribosomal RNA gene database project: improved data processing and web-based tools. Nucleic Acids Res. (2013) 41:D590–6. 10.1093/nar/gks1219 23193283 PMC3531112

[37] Davis NM Proctor DM Holmes SP Relman DA Callahan BJ. Simple statistical identification and removal of contaminant sequences in marker-gene and metagenomics data. Microbiome. (2018) 6:226. 10.1186/s40168-018-0605-2 30558668 PMC6298009

[38] McMurdie PJ Holmes S. phyloseq: an R package for reproducible interactive analysis and graphics of microbiome census data. PLoS ONE. (2013) 8:e0061217. 10.1371/journal.pone.0061217 23630581 PMC3632530

[39] Welch BL. The generalization of ‘student's' problem when several different population variances are involved. Biometrika. (1947) 34:28–35. 10.1093/biomet/34.1-2.28 20287819

[40] Shapiro SS Wilk MB. An analysis of variance test for normality (complete samples). Biometrika. (1965) 52:591–611. 10.1093/biomet/52.3-4.591

[41] Oksanen J Simpson GL Blanchet FG Kindt R Legendre P Minchin PR. vegan: Community Ecology Package. (2022). Available at: https://github.com/vegandevs/vegan (accessed October 11, 2022).

[42] Fernandes AD Macklaim JM Linn TG Reid G Gloor GB. ANOVA-like differential expression (ALDEx) analysis for mixed population RNA-Seq. PLoS ONE. (2013) 8:e67019. 10.1371/journal.pone.0067019 23843979 PMC3699591

[43] Fernandes AD Reid JN Macklaim JM McMurrough TA Edgell DR Gloor GB. Unifying the analysis of high-throughput sequencing datasets: characterizing RNA-seq, 16S rRNA gene sequencing and selective growth experiments by compositional data analysis. Microbiome. (2014) 2:15. 10.1186/2049-2618-2-15 24910773 PMC4030730

[44] Gloor GB Macklaim JM Fernandes AD. Displaying variation in large datasets: plotting a visual summary of effect sizes. J Comput Graph Stat. (2016) 25:971–9. 10.1080/10618600.2015.1131161

[45] McGovern KC Nixon MP Silverman JD. Addressing erroneous scale assumptions in microbe and gene set enrichment analysis. PLoS Comput Biol. (2023) 19:e1011659. 10.1371/journal.pcbi.1011659 37983251 PMC10695402

[46] Jeon SJ Galvão KN. An advanced understanding of uterine microbial ecology associated with metritis in dairy cows. Genomics Inform. (2018) 16:e21. 10.5808/GI.2018.16.4.e21 30602082 PMC6440669

[47] Bicalho MLS Machado VS Higgins CH Lima FS Bicalho RC. Genetic and functional analysis of the bovine uterine microbiota. Part I: Metritis versus healthy cows. J Dairy Sci. (2017) 100:3850–62. 10.3168/jds.2016-12058 28259404

[48] Chirino-Trejo JM Prescott JF. The identification and antimicrobial susceptibility of anaerobic bacteria from pneumonic cattle lungs. Can J Comp Med. (1983) 47:270–275. 6640410 PMC1235937

[49] Basbas C Garzon A Schlesener C van Heule M Profeta R Weimer BC. Unveiling the microbiome during post-partum uterine infection: a deep shotgun sequencing approach to characterize the dairy cow uterine microbiome. Anim Microbiome. (2023) 5:59. 10.1186/s42523-023-00281-5 37986012 PMC10662892

[50] Kronfeld H Kemper N Hölzel CS. Vaginal and uterine microbiomes during puerperium in dairy cows. Agriculture. (2022) 12:405. 10.3390/agriculture12030405

[51] VanRaden PM Sanders AH Tooker ME Miller RH Norman HD Kuhn MT. Development of a national genetic evaluation for cow fertility. J Dairy Sci. (2004) 87:2285–92. 10.3168/jds.S0022-0302(04)70049-1 15328243

