# Corrigendum: Use of intrauterine dextrose as an alternative to systemic antibiotics for treatment of clinical metritis in dairy cattle: a microbiome perspective

**DOI:** 10.3389/fvets.2025.1596455

**Published:** 2025-04-02

**Authors:** Jennine Lection, Emily Van Syoc, Asha Miles, Julia Hamilton, Marcela Martinez, Santiago Bas, Justin Silverman, Adrian Barragan, Erika Ganda

**Affiliations:** ^1^Intergraduate Degree Program in Integrative and Biomedical Physiology, Huck Institutes of the Life Sciences, The Pennsylvania State University, University Park, PA, United States; ^2^Department of Animal Science, College of Agricultural Sciences, The Pennsylvania State University, University Park, PA, United States; ^3^One Health Microbiome Center, The Pennsylvania State University, University Park, PA, United States; ^4^Department of Biology, Eberly College of Science, The Pennsylvania State University, University Park, PA, United States; ^5^Animal Genomics and Improvement Laboratory, Agricultural Research Service, United States Department of Agriculture, Beltsville, MD, United States; ^6^Department of Veterinary and Biomedical Sciences, College of Agricultural Sciences, The Pennsylvania State University, University Park, PA, United States; ^7^Phytobiotics Futterzusatzstoffe GmbH Bvd Villa Maria Córdoba Argentina, Villa Maria, Argentina; ^8^College of Information Sciences and Technology, The Pennsylvania State University, University Park, PA, United States; ^9^Department of Medicine, The Pennsylvania State University, Hershey, PA, United States; ^10^Institute for Computational and Data Science, The Pennsylvania State University, University Park, PA, United States

**Keywords:** antibiotic alternative, clinical metritis, dairy cattle, intrauterine dextrose, microbiome

In the published article, there was an error in the **Funding statement**.

One of the numbers for graduate student support was inadvertently left off of the publication in the original text statement: “For graduate student support, this publication was supported, in part, by NIH Grant T32GM108563 and by AFRI Predoctoral Fellowship [grant no. 2022-67011-36461] from the USDA National Institute of Food and Agriculture.” The correct Funding statement appears below.

